# Mitochondrial tRNA-Derived Fragments as Candidate Metastasis-Modifying RNA

**DOI:** 10.1158/2767-9764.CRC-26-0360

**Published:** 2026-08-28

**Authors:** Katy L. Swancutt, R. McKinnon Walsh, Sydney Quijano, Emily Schueddig, Devin C. Koestler, Adam D. Scheid, Tony Vanden Bush, Yi Jing, Isidore Rigoutsos, Danny R. Welch

**Affiliations:** 1 Department of Cancer Biology, The University of Kansas Medical Center, Kansas City, Kansas.; 2 The University of Kansas Cancer Center, Kansas City, Kansas.; 3 Department of Biostatistics and Data Science, The University of Kansas Medical Center, Kansas City, Kansas.; 4 Syntherna, Inc., Iowa City, Iowa.; 5 Computational Medicine Center, Thomas Jefferson University, Philadelphia, Pennsylvania.; 6 Department of Pathology and Laboratory Medicine, The University of Kansas Medical Center, Kansas City, Kansas.; 7 Department of Internal Medicine - Hematology and Oncology, The University of Kansas Medical Center, Kansas City, Kansas.; 8 Department of Cell Biology and Physiology, The University of Kansas Medical Center, Kansas City, Kansas.

## Abstract

How mitochondrial DNA (mtDNA) polymorphisms influence complex phenotypes remains poorly understood. Using mitochondrial–nuclear exchange mice, we previously showed that mtDNA single-nucleotide polymorphisms (SNP) modify metastasis, cardiovascular disease, and epigenetic marks independently of metabolic differences. The only mtDNA SNP correlating with these phenotypes resides in the gene encoding mitochondrial transfer RNA (tRNA)-arginine [*mt-tRNA*^*Arg*^* (UCG)*, *mt-TR*], suggesting a role for non–protein-coding loci. In this study, we identify and preliminarily characterize previously undescribed tRNA-derived fragments (tRF) generated from mt-TRs. Northern blotting revealed distinct tRF that are differentially expressed among mtDNA SNPs, between lung and liver, and between sexes. Surprisingly, small RNA sequencing of untreated RNA did not detect the same tRFs in high abundance. However, demethylating and restoring 5′-OH and 3′-PO_4_ termini allowed detection of sequences consistent with the northern blot bands. Enforcing exact matching to the mitochondrial genome and normalizing to their parental molecule revealed putative tRF sequences with shared cleavage sites. Based on connections among mtDNA SNPs, the resulting SNP-dependent tRF, and SNP–metastasis correlation, we propose that these tRFs may function as metastasis modifiers. These data also expand the functional output of the mitochondrial genome that can contribute to phenotype modification.

**Significance:**

This study describes identification and initial characterization of mitochondrial tRNA fragments that are suspected to regulate metastasis efficiency.

## Introduction

Hunter and colleagues (1) demonstrated that genetic background influences metastasis efficiency by crossing male MMTV-PyMT mice on an FVB/N background with females from distinct inbred strains. We hypothesized that maternally derived heritable modifiers from mitochondrial DNA (mtDNA) could be, at least partly, responsible for their observations (2–4). To isolate mitochondrial genotype as an independent experimental variable, mitochondrial–nuclear exchange (MNX) mice were generated (5, 6), enabling systematic evaluation of mtDNA contributions to tumor phenotypes. Mitochondrial polymorphisms exerted intrinsic and extrinsic changes in tumor and metastasis formation in an oncogenic driver-dependent manner (3, 4).

Accumulating data from experimental and clinical samples have added evidence that mtDNA affects multiple complex phenotypes (2, 6–11); however, the mechanism(s) responsible remain unknown. Due to mitochondria’s integral role in metabolism and because the mitochondrial genome encodes only 13 proteins, all in the electron transport machinery, metabolomic analysis of >700 metabolites was conducted. No global metabolic shifts in MNX mouse lung or mammary tissue attributable to mtDNA variation were observed. As one would expect, metabolites clustered predominantly by nuclear genotype (Supplementary Fig. S1). These null findings with respect to broad metabolic rewiring suggest that mtDNA may influence metastasis through mechanisms beyond canonical bioenergetics (3, 4). Coupled with limited, discrete changes at sites of nuclear DNA methylation (8), we hypothesized that non–protein-coding transcripts of the mitochondrial genome may function as metastasis modifiers.

**Figure 1.**
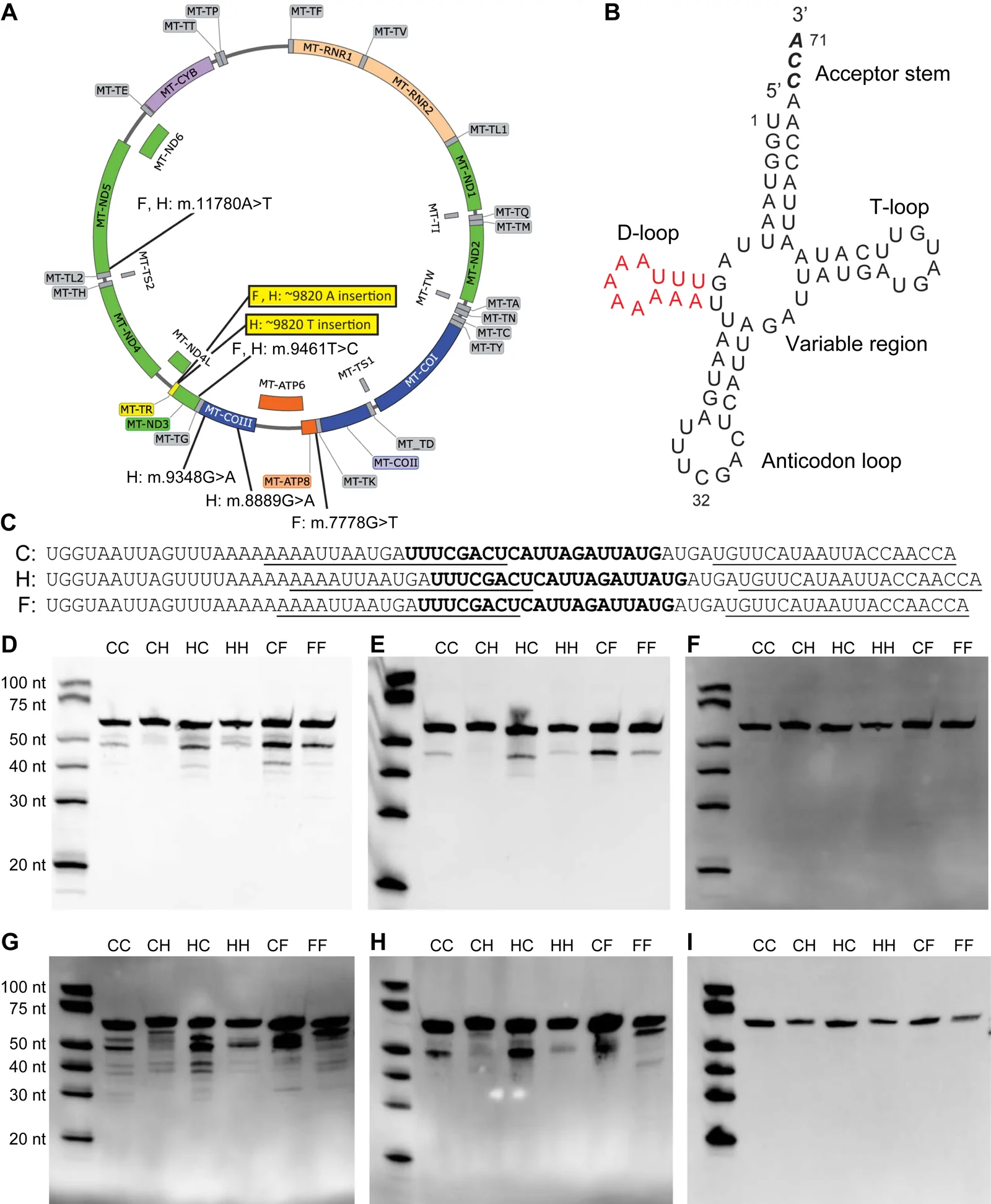
SNPs across mitochondrial genomes of MNX and wild-type mice correspond to tRF patterns in liver tissue. **A,** Relative to the C57BL/6J mouse mitochondrial genome (NC005089.1), SNPs in coding positions 7778, 8889, 9348, 9461, and 11780 and within the gene encoding tRNA^Arg^ (mt-TR) exist in the genomes of C3H/HeJ (CM004255.1) and FVB/NJ (CM004184.1). **B,** Across strains, SNPs at or near nucleotide position 9820 are within the *mt-TR* D-loop (red). **C,** Full sequences of *mt-TR* in C (C57BL/6J), H (C3H/H3J), and F (FVB/NJ) strains, written from 5′ to 3′ and including posttranscriptionally added 3′ terminal Cytosine-Cytosine-Adenine (CCA) caps. Regions targeted by northern blot hybridization probes are indicated as follows: The mid-molecule underlined region is bound by Probe R3, the region in bold is bound by Probe R4, and the underlined region at the far 3′ end is bound by Probe R5. **D–I,***mt-TR* at 71–73 nt in length, depending on mtDNA strain (note connecting lines in **D**), and tRF detected via Northern blotting using Probe R3 (**D** and **G**), R4 (**E** and **H**), or R5 (**F** and **I**) in male (**D–F**) or female (**G–I**) liver tissue.

Comparative analysis of single-nucleotide polymorphisms (SNP) across MNX and wild-type mitochondrial genomes highlighted a polymorphic locus with strain-dependent insertions and deletions within the mitochondrial transfer RNA (tRNA) for arginine [*mt-tRNA*^*Arg*^* (UCG)*, hereafter abbreviated *mt-TR*; Fig. 1A–C]. We hypothesized that SNPs in *mt-TR* modulate the generation of tRNA cleavage products—small noncoding RNAs called tRNA-derived fragments (tRF; refs. 12–14)—which may influence biological processes necessary for metastasis. Likewise, tRFs containing different SNPs could differentially influence phenotypes.

tRFs have emerged as a class of regulatory small RNAs generated from precise cleavage of mature or precursor tRNA (12–14). Characterized primarily from nuclear-encoded tRNA, tRFs have been implicated in translational repression (15), RNA silencing (16), modulation of apoptosis (17), and cancer progression and metastasis (18–20). Despite growing appreciation of nuclear tRF biology, the mitochondrial genome represents an underexplored source of small regulatory RNAs. In contrast to nuclear tRNA genes, mitochondrial tRNAs are transcribed as part of polycistronic transcripts, lack introns, and are processed according to the tRNA punctuation model, raising the possibility that distinct biogenesis pathways could generate unique mitochondrial tRFs (21–25).

Here, we report the discovery and early characterization of previously undescribed tRFs derived from a SNP-containing mitochondrial tRNA. Based on connections among mtDNA SNPs, the resulting SNP-dependent tRFs, and SNP–metastasis correlation, we propose that these candidate tRFs may function as metastasis modifiers. These findings expand the repertoire of mitochondrial gene products and suggest a new framework through which mtDNA variation may influence cancer progression in both mouse models and human disease.

## Materials and Methods

### Animals

MNX mouse lines were generated as previously described by pronuclear transfer between fertilized oocytes of defined inbred strains to create mice with mismatched nuclear and mitochondrial genomes (5, 26). MNX colonies were bred in-house and included lineage combinations from C57BL/6J (RRID:IMSR_JAX:000664), C3H/HeN (RRID:MGI:2160972), FVB/NJ (RRID:IMSR_JAX:001800), and BALB/cJ (RRID:MGI:2161072) backgrounds, with wild-type controls obtained from commercial vendors (The Jackson Laboratory or Inotiv, formerly Envigo) and acclimated after shipping for 7 days or more. We represent MNX strains using a two-letter code in which the first letter indicates the nuclear genome strain of origin and the second letter indicates the mtDNA strain (C = C57BL/6J; H = C3H/HeN; F = FVB/NJ). Genotyping was conducted using the Extract-N-Amp Tissue PCR Kit (MilliporeSigma, XNAT2) and GoTaq Green Master Mix (Promega, M712B). Mitochondrial backgrounds of amplicons were verified via restriction fragment length polymorphism analysis of strain-specific mitochondrial SNPs, including loci at positions 9461 and 9348, using BclI (New England Biolabs, R0160S) or PflFI (New England Biolabs, R0595S) digestion, as previously described (3–6). Lung and liver tissues were collected from healthy adult animals, between 5 and 15 weeks of age, maintained using standard husbandry practices, and euthanized via cervical dislocation confirmed by bilateral thoracotomy. All animal procedures were approved by the Institutional Animal Care and Use Committee at the University of Kansas Medical Center.

### Small RNA extraction

Tissues were preserved in RNAlater (Invitrogen, AM7024) and frozen prior to RNA extraction. Small RNA was isolated using the mirVana miRNA Isolation Kit (Thermo Fisher Scientific, AM1560) according to the manufacturer’s instructions, with one exception: Rather than being discarded, the corresponding large RNA fractions of small RNA isolations were retained, washed, and eluted to assess RNA integrity by visualization of 28S and 18S rRNA bands following agarose gel electrophoresis. Intact RNA samples were pooled by sodium acetate–propanol precipitation and washed in 70% ethanol, followed by resuspension in nuclease-free water. RNA concentration was quantified using the Qubit microRNA Assay Kit (Thermo Fisher Scientific, Q32881).

### Northern blotting

Northern blotting was performed using denaturing urea-PAGE. Pooled small RNA (5 μg per lane) was denatured at 70°C for 5 minutes prior to loading, resolved on 15% 8 mol/L urea-PAGE gels, and electrophoretically transferred to nylon membranes. Membranes were UV-cross-linked and prehybridized in ULTRAhyb buffer (Invitrogen, AM8663) prior to overnight 37°C hybridization with 3′ end digoxigenin (DIG)-labeled locked nucleic acid (ssDNA) probes, each custom synthesized by Integrated DNA Technologies. The following probe sequences were tested and named for complementary regions of *mt-TR* from 5′ to 3′: R1a: TTTTTAAACTAATTACCA, R1b (specific to C3H-origin mtDNA products): TTTTAAAACTAATTACCA, R2: AATCATTAATTTTTTTT, R3: AGTCGAAATCATTAATTTT, R4: CATAATCTAATGAGTCGAAA, and R5: TGGTTGGTAATTATGAACA. After high- and low-stringency saline sodium citrate/SDS washes, membranes were blocked (Roche, 11585762001) and incubated with alkaline phosphatase–conjugated anti-DIG antibody (Roche, 0335375910). Chemiluminescent signal was detected via digital imaging (Azure 600; RRID:SCR_023780) after application of CDP-Star substrate (Sigma, C0712). Exposure times (5-minute manual) were used for imaging all blots presented to facilitate comparisons. A DIG-labeled RNA ladder conjugated to a blue color marker was used to approximate tRNA and tRF sizes (DiagnoCine, FNK-DM270). The quality of pooled RNA samples was assessed in separate blots using a probe for 5S rRNA (AAGCCTACAGCACCCGGTATT). The full-length *mt-TR*, considered a housekeeping transcript, served as a convenient loading control. All experiments were conducted in duplicate or triplicate; representative blots are shown.

### Pretreatment of small RNA samples and RNA sequencing

RNA isolated from mouse lungs using the mirVana miRNA Isolation Kit was initially submitted to RealSeq for exploratory tRNA fragment sequencing using a single-adapter circularization-based library preparation approach (27). For subsequent experiments, RNA was extracted from mouse liver, pooled, and pretreated using the rtStar tRF&tiRNA Pretreatment Kit (Arraystar, AS-FS-005) to remove RNA modifications thought to interfere with tRF detection, including 3′-aminoacyl groups, 2′,3′-cyclic phosphates, and 5′ hydroxyl termini, as well as 1-methyladenosine (m^1^A), 1-methylguanosine, and 3-methylcytidine methylation. Following pretreatment, RNA quality and concentration were assessed using the Agilent TapeStation 2400 with RNA ScreenTape and High Sensitivity RNA ScreenTape assays. Small RNA libraries were prepared using the QIAseq miRNA UDI Library Kit and indexed with the QIAseq UDI miRNA Index Kit (QIAGEN). Libraries were normalized, pooled, and sequenced on a NovaSeq instrument by the University of Kansas Medical Center Genomics Core Facility.

### RNA sequencing preprocessing and alignment

Read quality was assessed using FastQC (v0.12.1, RRID:SCR_014583). unique molecular modifier (UMI)-tools (v1.1.6, RRID:SCR_017048) was used for UMI and read extraction; then Cutadapt (v5.0, RRID:SCR_011841) was used to retain reads with a minimum read length of 16 base pairs and a maximum of 100 base pairs. Bowtie (v1.3.1, RRID:SCR_005476) was used for mapping with parameters set for no gaps and no mismatches. Reads were deduplicated using UMI-tools and aligned to the genomic sequence of mature mitochondrial *mt-TR*. As there are known SNPs in the *mt-TR* sequence for different mouse strains, each sample was mapped to its respective reference sequence (Fig. 1C). The resulting set of aligned reads were mapped to the full mouse genome with the UCSC mm10 assembly as the reference using the Bowtie aligner. If the sequence mapped to another location, it was marked as a false positive and removed from downstream analysis. The number of reads for each unique sequence was extracted for each sample.

### Analysis

Read counts of the putative tRF sequences were normalized by dividing the number of reads of a unique sequence by the total number of mapped reads to *mt-TR* and multiplying by 1,000,000 (Eq. A).

$$\begin{array}{c} mt-TR-Normalized\;Reads\;=\;\frac{Total\;Read\;Count\;of\;tRF}{Total\;Mapped\;Reads\;to\;mt‐TR\;}\;\times 10^{6} \end{array}$$ (A)

From lists of the top 10 most abundant *mt-TR* aligned reads (listed by sample in Supplementary Table S1), candidate tRF sequences were tentatively nominated based on average normalized reads by mitochondrial strain of origin and in accordance with bands visualized in the gold standard Northern blots (Table 1). The mouse tissue atlas of small noncoding RNA (GSE119661) was used for comparison of *mt-TR*–aligned sequences in publicly available data (28).

**Table 1.** Candidate tRF sequences aligned to *mt-TR* determined via RNA sequencing.

Strains	Fragment sequence	Average *mt-TR*–normalized reads
CC and HC	TGGTAATTAGTTTAAAAAAAATTAATGATTTCGACTCATTAGATTATGATGA	172,576.40
	TGGTAATTAGTTTAAAAAAAATTAATGATTTCGACTCATTAGA	120,712.30
	TTTAAAAAAAATTAATGATTTCGACTCATTAGATTATGATGA	57,703.44
	TGGTAATTAGTTTAAAAAAAATTAATGATTTCGACTCATTAGATT	23,131.11
	TGGTAATTAGTTTAAAAAAAATTAATGATTTCGACTCATTAGATTATGA	10,323.80
	TTAGTTTAAAAAAAATTAATGATTTCGACTCATTAGATTATGATGA	9,487.48
	AAAAAAAATTAATGATTTCGACTCATTAGATTATGATGATGTTCATAATTACCAACCA	8,737.05
	TTTAAAAAAAATTAATGATTTCGACTCATTAGATTATGATGATGTTCATAATTACCAACCA	8,506.63
	TGGTAATTAGTTTAAAAAAAATTAATGATTTCGACTCATT	8,185.45
	TTTCGACTCATTAGATTATGATGATGTTCATAATTACCAACCA	6,754.55
HH and CH	TGGTAATTAGTTTTAAAAAAAAATTAATGATTTCGACTCATTAGATTATGATGA	31,224.23
	TGGTAATTAGTTTTAAAAAAAAATTAATGATTTCGACTCATTAGA	22,326.65
	TTTCGACTCATTAGATTATGATGATGTTCATAATTACCAACCA	13,141.58
	TTTTAAAAAAAAATTAATGATTTCGACTCATTAGATTATGATGA	13,050.23
	TTCGACTCATTAGATTATGATGATGTTCATAATTACCAACCA	10,700.44
	TTTAAAAAAAAATTAATGATTTCGACTCATTAGATTATGATGATGTTCATAATTACCAACCA	10,510.03
	AAAAAAAAATTAATGATTTCGACTCATTAGATTATGATGATGTTCATAATTACCAACCA	10,249.75
	TAAAAAAAAATTAATGATTTCGACTCATTAGATTATGATGATGTTCATAATTACCAACCA	9,618.80
	TTTTAAAAAAAAATTAATGATTTCGACTCATTAGATTATGATGATGTTCATAATTACCAACCA	8,134.17
	TTAAAAAAAAATTAATGATTTCGACTCATTAGATTATGATGATGTTCATAATTACCAACCA	7,980.47
FF and CF	TGGTAATTAGTTTAAAAAAAAATTAATGATTTCGACTCATTAGATTATGATGA	241,040.50
	TGGTAATTAGTTTAAAAAAAAATTAATGATTTCGACTCATTAGA	96,766.04
	TTTAAAAAAAAATTAATGATTTCGACTCATTAGATTATGATGA	41,784.64
	TGGTAATTAGTTTAAAAAAAAATTAATGATTTCGACTCATTAGATT	16,122.85
	TGGTAATTAGTTTAAAAAAAAATTAATGATTTCGACTCATTAGATTATGA	14,831.61
	TTTAAAAAAAAATTAATGATTTCGACTCATTAGATTATGATGATGTTCATAATTACCAACCA	9,913.05
	TGGTAATTAGTTTAAAAAAAAATTAATGATTTCGACTCATTAGATTATGATGATG	9,153.11
	TTAGTTTAAAAAAAAATTAATGATTTCGACTCATTAGATTATGATGA	9,045.27
	TGGTAATTAGTTTAAAAAAAAATTAATGATTTCGACTCATTA	8,519.76
	TTTCGACTCATTAGATTATGATGATGTTCATAATTACCAACCA	8,355.08

NOTE: tRF sequences from pretreated small RNA samples from female mouse liver ranked in decreasing order of reads normalized to reads of *mt-TR*. *mt-TR*–normalized reads were averaged across replicates among strains with the same mtDNA genome and filtered to include putative tRF sequences corroborating northern blot results based on size (Fig. 1G–I).

### tRNA structure modeling

Sequences for *mt-TR* in each strain (C: C57BL/6J, H: C3H/HeJ, F: FVB/NJ) were input into AlphaFold3 (29, 30) to predict potential changes in structure due to SNPs across strains. Due to low confidence in unmodified structure prediction, known human *mt-TR* modifications including m^1^A at positions 9 and 16, 2N-methylguanosine (m^2^G) at position 10, and pseudouridine (Ψ) at positions 29 and 36 (31) were included in the modeling to increase the confidence of folding predictions.

## Results

### SNPs in the *mt-TR* gene correlate with tRF generation

Despite the small size of the mitochondrial genome, polymorphisms influence diverse biological processes, including metastatic progression (3, 4, 11), potentially mediated by tRFs (3, 4, 8–11, 19). Because tRFs represent a distinct, and increasingly recognized, class of regulatory small RNA, we examined whether *mt-TR* might similarly give rise to previously uncharacterized RNA species. Using Northern blotting and focusing initially on tissues in which metastases often arise, we identified discrete patterns of RNA bands derived from *mt-TR* in male mouse liver (Fig. 1D–F). These fragments were detected under basal conditions and were not assessed under conditions of cellular stress.

**Figure 2.**
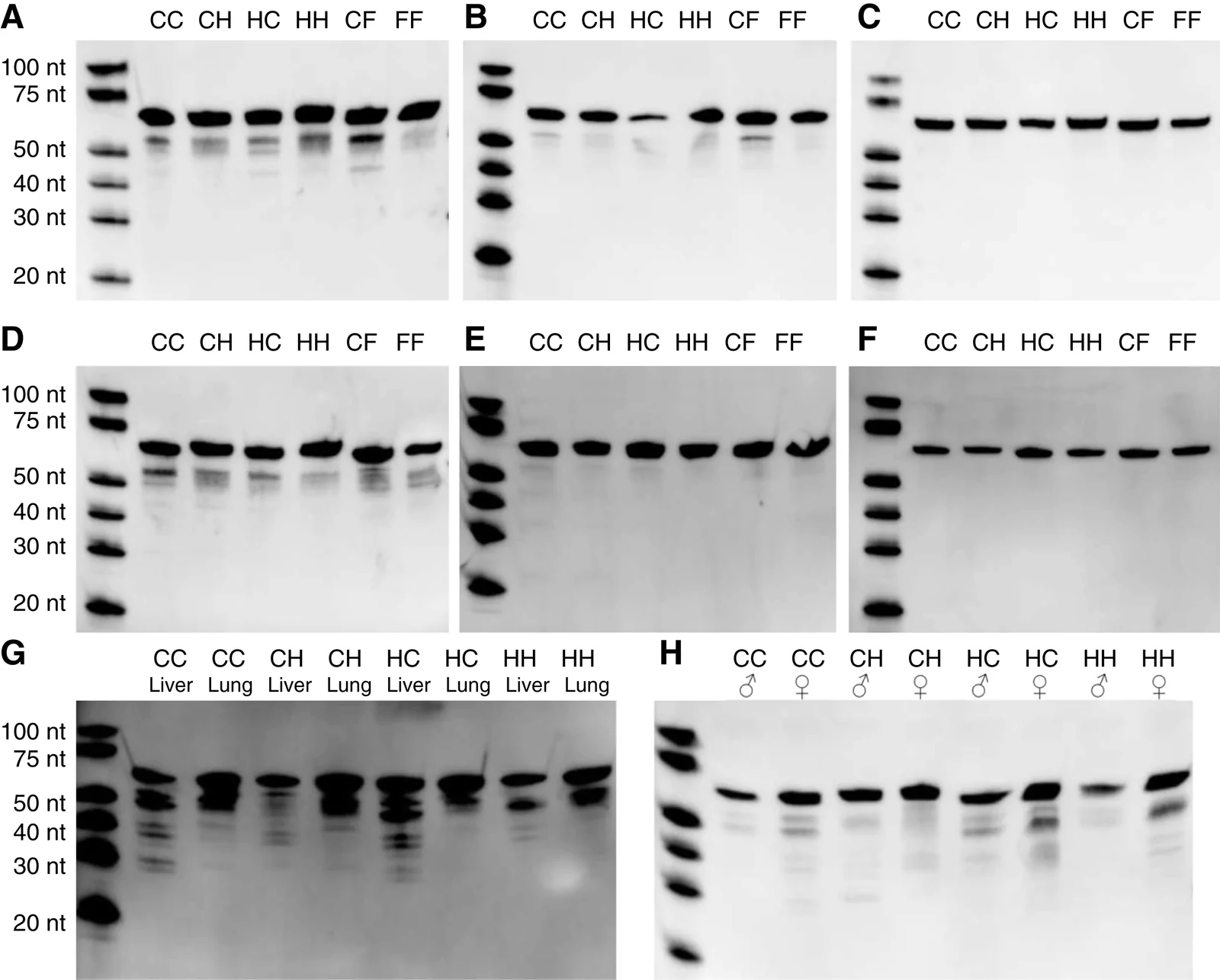
tRF patterns are consistent across probed regions but differ by tissue and sex. **A–F,***mt-TR* (71–73 nt in length) and tRF detected via Northern blotting using Probe R3 (**A** and **D**), R4 (**B** and **E**), or R5 (**C** and **F**) in male (**A–C**) or female (**D–F**) lung tissue. **G,** Side-by-side comparison of tRF patterns in female liver vs. lung tissues. **H,** Side-by-side comparison of tRF patterns in liver tissue of male vs. female mice.

Using a hybridization probe designed to capture the most likely expected tRF species, internal tRF or “i-tRF” (25), we observed the greatest diversity of tRF bands when targeting the mid-region of *mt-TR*, complementary to positions 18 to 36 in CC and HC strains, 19 to 37 in FF and CF strains, and 20 to 38 in HH and CH strains (probe R3, Fig. 1C). Importantly, banding patterns were more similar among strains sharing the same mitochondrial genome (best visualized in Fig. 1D), indicating that mtDNA background influences fragment abundance and/or processing. Several fragments remained detectable when the hybridization probe target region was shifted 11 nt toward the 3′ end (probe R4, Fig. 1E). Species smaller than ∼50 nt were also consistently observed. In contrast, hybridization against the 3′ end of *mt-TR* yielded no detectable fragments (probe R5, Fig. 1F). Similar tRF species were detected in female liver (Fig. 1G–I) as well as male (Fig. 2A–C) and female lung (Fig. 2D–F). Across tissues and sexes, probing the 3′ terminus of *mt-TR* unexpectedly failed to detect corresponding small RNAs (24). However, our findings reinforced the possibility that stable fragments arising from the 5′ and internal regions of the transcript (“5′-tRF” according to established nomenclature; ref. 25) are produced. Band intensities suggest fragment abundance may vary by mitochondrial genotype, tissue, and sex (directly compared in Fig. 2G and H).

Taken together, the predominant tRF species observed at approximately 50 nt in size is consistent with the removal of roughly 20 nt from the 3′ terminus. Consisting of the 5′ and middle aspects of *mt-TR*, this 50 nt moiety is larger than the canonical 5′ half tRF species commonly detected by RNA sequencing platforms. The absence of detectable bands representing 19 to 21 nt fragments corresponding to any potential 3′ cleavage product suggests rapid degradation or that the abundance is below the limits of detection in our northern blotting protocol. Nonetheless, such products can be amplified and sequenced via miRNA-specific library preparation (Supplementary Table S1). Based on these findings, we propose a model in which mtDNA SNPs alter mt-TR structure, potentially influencing nuclease accessibility and cleavage patterns during tRF biogenesis, consistent with the strain-specific differences in fragment patterns and abundance observed by Northern blotting and correlating with mtDNA-dependent metastatic burden.

### tRF derived from *mt-TR* are posttranscriptionally modified and cleaved at specific sites

Attempting to define the sequences of tRF cleaved from *mt-TR*, we first performed small RNA sequencing using single-adapter and circularization-based library preparation approaches previously validated for miRNA profiling (27). This initial effort failed to detect *mt-TR*-aligned fragments of appropriate size corresponding to the bands visualized via northern blotting (data accessible at GSE324706). tRNA is extensively modified, harboring methylated bases and other chemical alterations (31). Cleavage by endoribonucleases (e.g., angiogenin, members of the RNase T2 or RNase A superfamilies) may generate fragments bearing noncanonical 5′ and/or 3′ termini (22, 32, 33). We therefore incorporated enzymatic correction of termini to restore 5′-phosphate and 3′-hydroxyl groups, along with demethylation of select nucleosides using an ArrayStar kit. We note that fragments containing additional uncorrected modifications may still evade detection. Quantitative interpretation of tRNA and tRF sequencing remains inherently constrained by multiple, interacting technical biases that affect both detection and apparent abundance.

Posttranscriptional modifications within tRNAs, such as m^1^A and m^2^G residues present in human *mt-TR* (31), can impede reverse transcription through premature termination, misincorporation, or variable readthrough, thereby distorting both recovery and sequence fidelity of tRF (34). Although demethylation strategies can partially alleviate these barriers, reaction efficiency is unknown and may introduce random RNA backbone cleavage via α-ketoglutarate and ferrous ion–mediated Fenton chemistry, which likely enriches shorter fragments independent of their biological origin (35). Furthermore, heterogeneity in tRF end chemistry, persistent secondary structure, and biases in adapter ligation, size selection, and PCR amplification collectively skew library composition in a sequence- and structure-dependent manner. Short read lengths also complicate unique alignment, particularly among highly homologous tRNA genes and mitochondrial–nuclear sequence paralogs, introducing ambiguity in read assignment (36). To improve confidence in predictions and better reflect known principles of tRNA folding (37), sequences for each strain were modeled according to reported post-transcriptional modifications (PTM) of human mt-TR (m^1^A, m^2^G; Ψ: pseudouridine). Together, these factors indicate that tRF abundance observed via RNA sequencing should be interpreted as a function of both biological signal and selective technical observability, rather than a strictly quantitative measure of intracellular RNA levels. Relative to direct observation via gold standard methods such as Northern blotting, it is highly plausible that RNA sequencing, with or without enzymatic pretreatment, underrepresents quantities of tRNA and tRF to an unspecified extent.

Following end correction and demethylation, only partial solutions to the complexity of tRNA and tRF RNA sequencing, several candidate fragments aligning uniquely to *mt-TR* were nominated (Supplementary Table S1). Although these fragment sequences can occasionally be found among publicly available data in mouse lung or liver, typical abundance (in reads per million) was marginally improved in pretreated data. When normalized to the reads of *mt-TR*, the most abundant species shared an identical cleavage site across strains despite underlying mtDNA SNPs, but total length was SNP-dependent (Table 1), supporting the conclusion that these RNAs are actively and reproducibly generated rather than degradation products, as RNA blotting would show a smear in the latter case.

**Figure 3.**
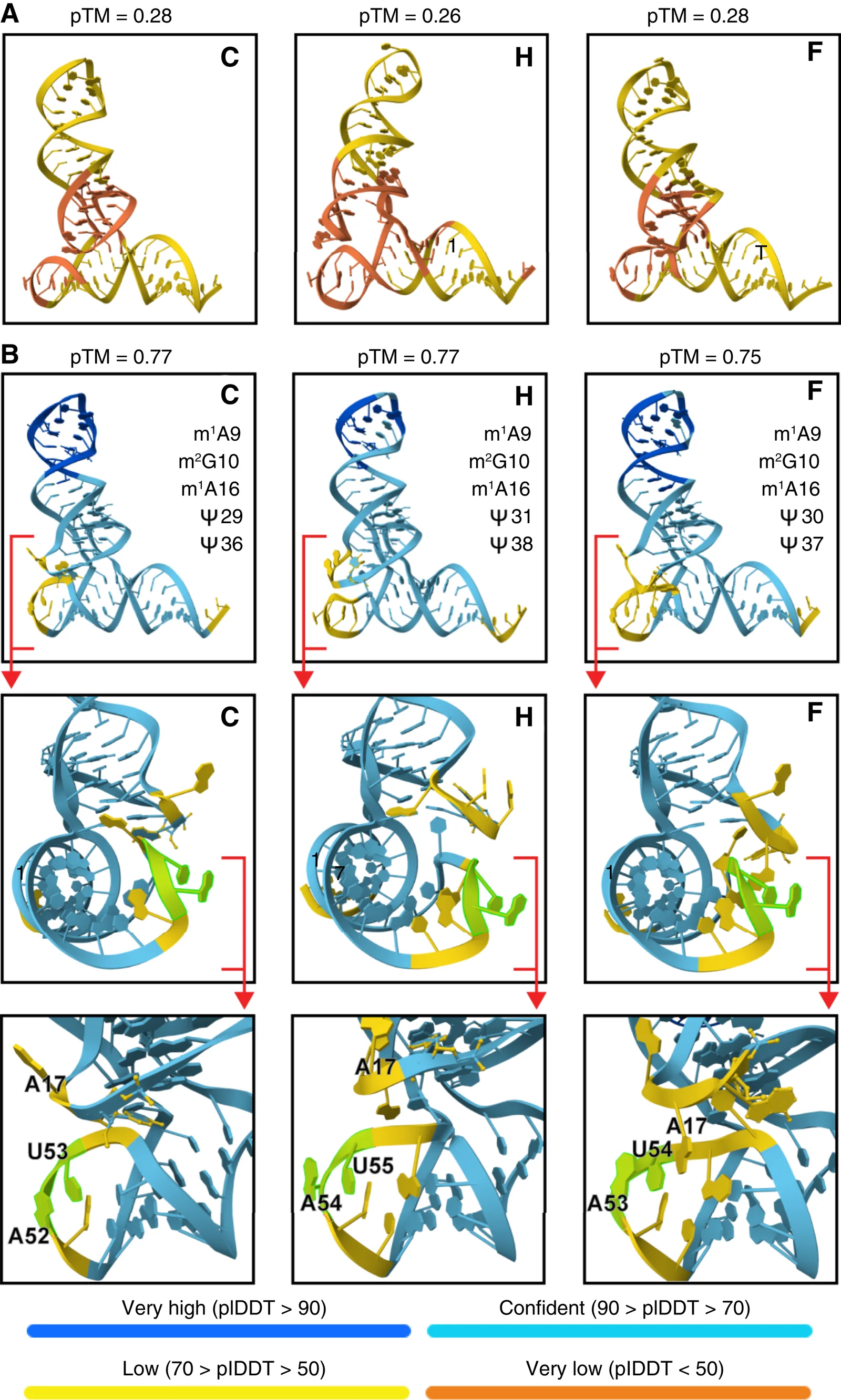
AlphaFold3 predictive modeling of *mt-TR* suggests different stability and D–T-loop structures among strains. **A,** Sequences for each *mt-TR* (from left to right: C, H, and F) modeled without posttranscriptional modifications (PTM) and displayed predicted instability/low confidence. Predicted template modeling “pTM” scores range from 0.26 to 0.28 on a scale of 0–1. **B,** Three rows of images are displayed with increased zoom from top to bottom. To improve confidence predictions and better reflect known principles of tRNA folding (37), sequences for each strain were modeled according to reported PTM of human *mt-TR* (m^1^A, m^2^G, and Ψ; ref. 31). PTM inclusion increased predicted stability and confidence scoring compared with that of unmodified sequences (pTM range, 0.75–0.77 vs. 0.26–0.28). The D–T-loop interactions have the lowest scoring confidence, which aligns with the commonly observed transcript end (Table 1) of fragments between C: U53/G54; H: U55/G56; F: U54/G55 (highlighted in green in the bottom two rows of images). Nucleotides and structure of predicted interactions between the D–T-loop differ across strains: T-loop G54 is predicted to interact with the D-loop in C mt-TR; T-loop U57 is predicted to interact with the D-loop in H *mt-TR*; both U54/G55 are predicted to interact specifically with A17 of the T-loop in C *mt-TR*. Specific predictive bonding and distances between the center of nucleotides are listed in tRF patterns in liver tissue. pIDDT, predicted local distance difference test.

Focusing on the proposed cleavage site of the predominant fragment, AlphaFold3 (29, 30) predictive modeling of *mt-TR* across all three strains displayed the lowest relative predicted stability/confidence surrounding this site in the region of D–T-loop interactions (Fig. 3A and B). Predicted structures of *mt-TR* demonstrate potential differential exposure and stability of adenine (52–54) and uracil (53–55) in the T-loop (Supplementary Fig. S2; Supplementary Table S2). We speculate that the proposed differential D–T-loop interaction of strain-specific *mt-TR* could contribute to stability or cleavage susceptibility at this site to be investigated in future studies.

## Discussion

Collectively, these findings establish the existence of previously uncharacterized mitochondrial tRFs in which the pattern of expression is influenced by mitochondrial genotype, tissue, and sex. The data support a model in which mtDNA polymorphisms modulate tRNA structure and cleavage, generating distinct small RNA species with potential regulatory activity. Elucidating the biogenesis, interacting partners, and functional consequences of these mitochondrial tRFs will be essential to understanding how mitochondrial genetic variation contributes to metastasis.

## Supplementary Material

Supplementary Figure 1 Metabolomic analysis of female MNX or WT mouse lung tissue.

Supplementary Figure 2 AlphaFold3 predictive modeling of D-T Loop interactions of mt-TR.

Supplementary Table 1 Top ten most abundant sequences aligning to mt-TR in each sample normalized to total mapped reads (in reads per million, RPM). Notable low overall abundance of sequences from pre-treated small RNA extracted from female mouse liver reflects technical challenges in the state of the art of RNA sequencing of tRNA and tRF (n = 3 samples per WT or MNX strain).

Supplementary Table 2 AlphaFold3 predictive modeling of D-T Loop interactions of mt-TR

## Data Availability

RNA sequencing data are deposited in the Gene Expression Omnibus (GEO; pretreated small RNA sequencing under GEO accession GSE324681; RealSeq small RNA sequencing under GEO accession GSE324706). Processed metabolomics data are accessible via an interactive heatmap at https://maayanlab.cloud/clustergrammer/viz_sim_mats/69a61efb1e6cf6050d0a600b/clustering%20input.txt. Images, metabolomics data, and other data are available from the authors upon request.
