# Supplementary material for: Mitochondrial tRNA-Derived Fragments as Candidate Metastasis-Modifying RNA
Source: Cancer Res Commun. 2026 Aug 28;6(8):2017–25. doi: 10.1158/2767-9764.CRC-26-0360 (PMC13522972; doi:10.1158/2767-9764.CRC-26-0360)
Supplement: Supplementary file 1 [file crc-26-0360_supplementary_figure_1_suppsf1.pdf]

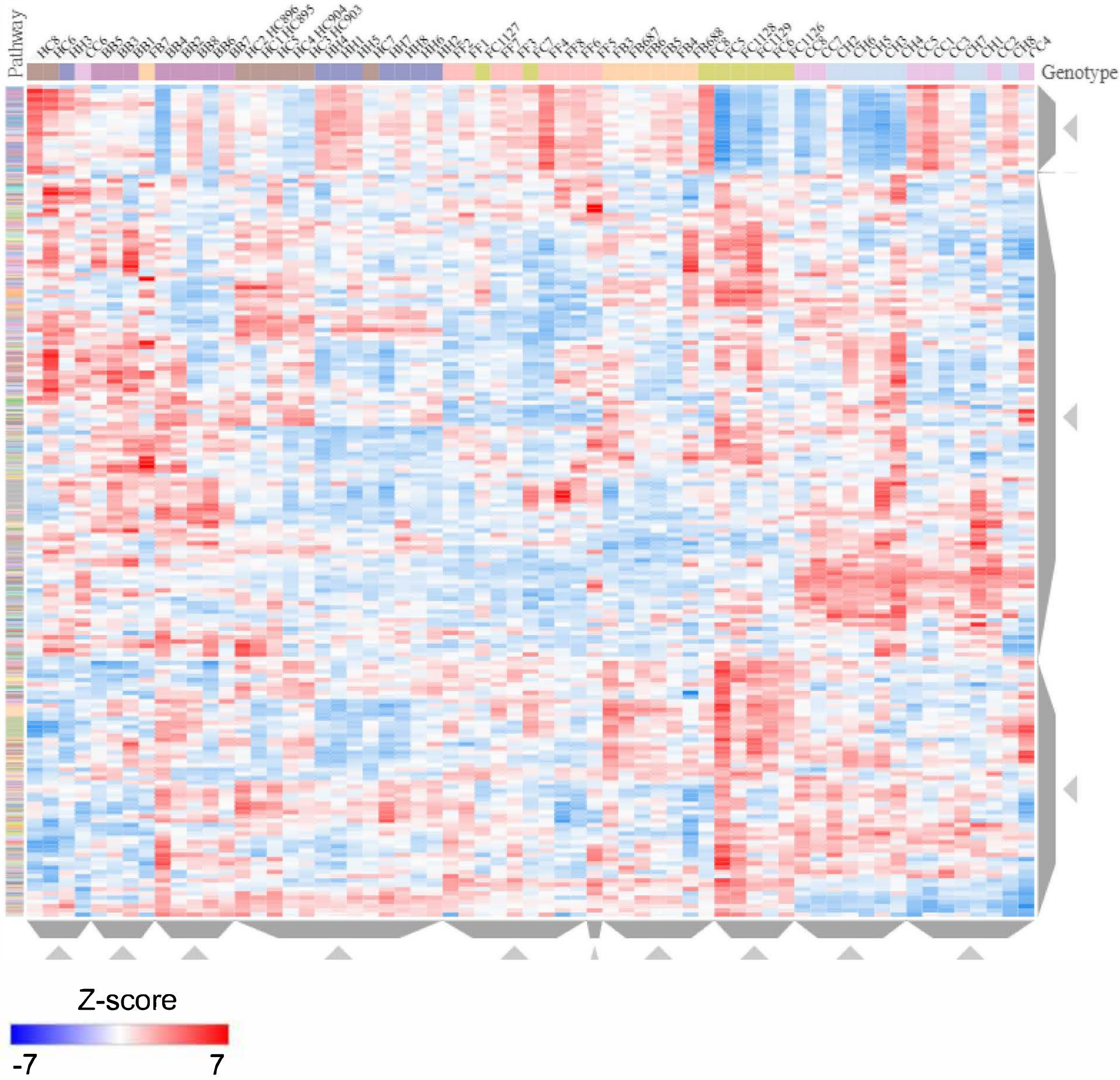

Supplementary Figure 1: Metabolomic analysis of female MNX or WT mouse lung tissue.
