# Supplementary material for: Mitochondrial tRNA-Derived Fragments as Candidate Metastasis-Modifying RNA
Source: Cancer Res Commun. 2026 Aug 28;6(8):2017–25. doi: 10.1158/2767-9764.CRC-26-0360 (PMC13522972; doi:10.1158/2767-9764.CRC-26-0360)
Supplement: Supplementary file 2 [file crc-26-0360_supplementary_figure_2_suppsf2.pdf]

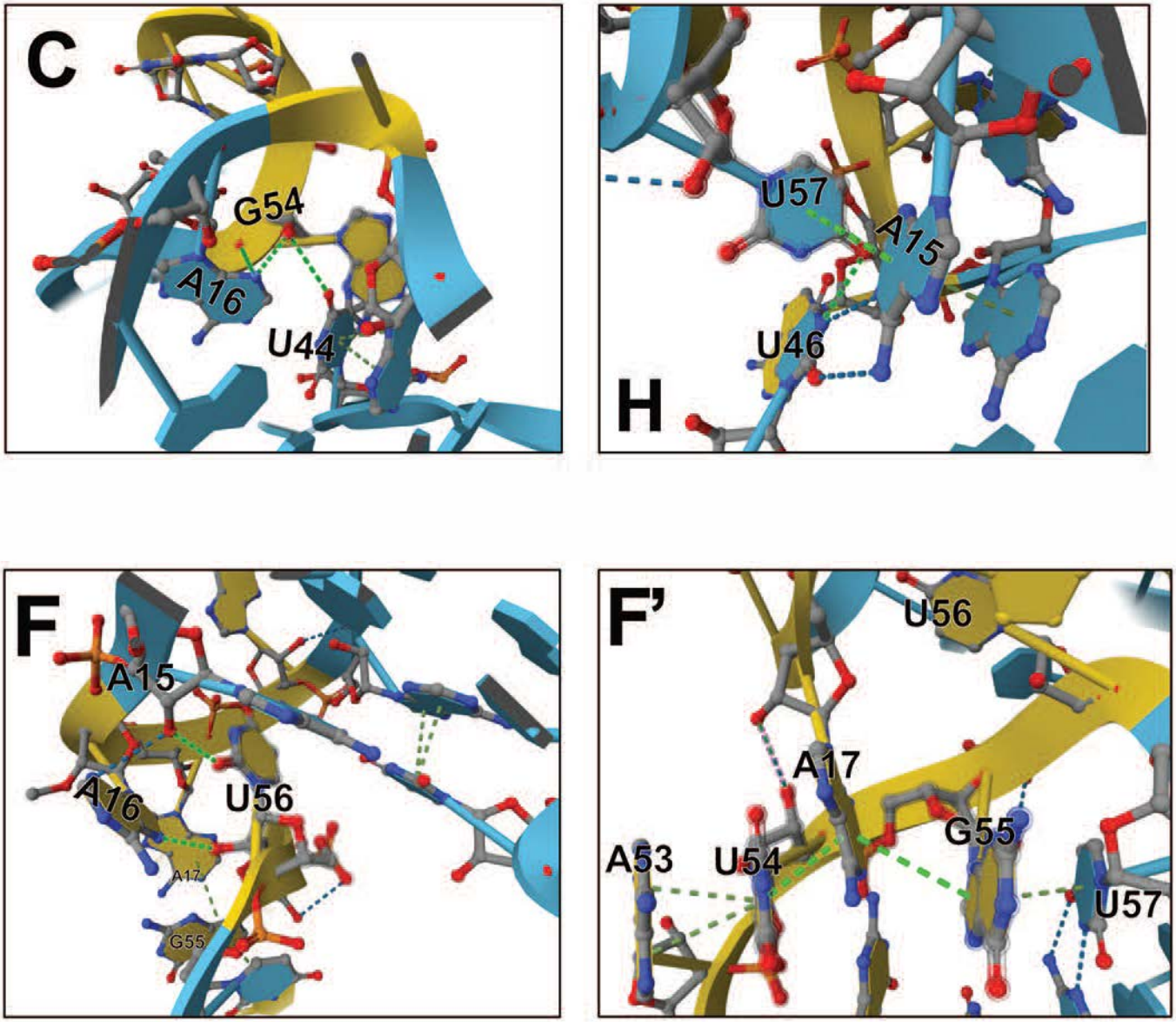

Supplementary Figure 2: AlphaFold3 predictive modeling of D-T Loop interactions of mt-TR.
