# Supplementary material for: Mitochondrial tRNA-Derived Fragments as Candidate Metastasis-Modifying RNA
Source: Cancer Res Commun. 2026 Aug 28;6(8):2017–25. doi: 10.1158/2767-9764.CRC-26-0360 (PMC13522972; doi:10.1158/2767-9764.CRC-26-0360)
Supplement: Supplementary file 3 [file crc-26-0360_supplementary_table_1_suppst1.docx]

**Supplementary Table 1: Top ten most abundant sequences aligning to mt-TR in each sample normalized to total mapped reads (in reads per million, RPM)**. Notable low overall abundance of sequences from pre-treated small RNA extracted from female mouse liver reflects technical challenges in the state of the art of RNA sequencing of tRNA and tRF (n = 3 samples per WT or MNX strain).

| Strain & Sample ID | Fragment Sequence | RPM |
| --- | --- | --- |
| CC 1 | -----------------------------------------------------TGTTCATAATTACCAACCA | 119.749 |
|  | TGGTAATTAGTTTAAAAAAAATTAATGATTTCGACTCATTAGATTATGATGA------------------- | 73.178 |
|  | ----------------------------------------------------TGTTCATAATTACCAACC- | 54.057 |
|  | TGGTAATTAGTTTAAAAAAAATTAATGATTTCGACTCATTAGA---------------------------- | 42.388 |
|  | ----------TTTAAAAAAAATTAATGATTTCGACTCATTAGATTATGATGA------------------- | 31.346 |
|  | ------------------------------------------------ATGATGATGTTCATAATTACCAACCA | 10.341 |
|  | -----------------------------------------------------GTTCATAATTACCAACCA | 8.959 |
|  | ------TTAGTTTAAAAAAAATTAATGATTTCGACTCATTAGA---------------------------- | 8.941 |
|  | ------TTAGTTTAAAAAAAATTAATGATTTCGACTCATTAGATTATGATGA------------------- | 8.582 |
|  | -------------------------------------------TTATGATGATGTTCATAATTACCAACCA | 8.312 |
| CC 2 | TGGTAATTAGTTTAAAAAAAATTAATGATTTCGACTCATTAGATTATGATGA------------------- | 89.689 |
|  | TGGTAATTAGTTTAAAAAAAATTAATGATTTCGACTCATTAGA---------------------------- | 51.260 |
|  | ----------TTTAAAAAAAATTAATGATTTCGACTCATTAGATTATGATGA------------------- | 28.771 |
|  | ----------------------------------------------------TGTTCATAATTACCAACCA | 25.210 |
|  | ----------------------------------------------------TGTTCATAATTACCAACC- | 8.616 |
|  | ------------------------------------------------ATGATGTTCATAATTACCAACCA | 8.367 |
|  | TGGTAATTAGTTTAAAAAAAATTAATGATTTCGACTCATTAGATT-------------------------- | 7.683 |
|  | -----------------------------------------------------GTTCATAATTACCAACCA | 6.361 |
|  | ---------------------------------------------------ATGTTCATAATTACCAACCA | 6.330 |
|  | --------------------------------------------------------CATAATTACCAACCA | 6.081 |
| CC 3 | TGGTAATTAGTTTAAAAAAAATTAATGATTTCGACTCATTAGATTATGATGA------------------- | 183.928 |
|  | TGGTAATTAGTTTAAAAAAAATTAATGATTTCGACTCATTAGA---------------------------- | 109.802 |
|  | TGGTAATTAGTTTAAAAAAAATTAATGATTTCGACTCATTAGATT-------------------------- | 28.775 |
|  | ----------TTTAAAAAAAATTAATGATTTCGACTCATTAGATTATGATGA------------------- | 13.405 |
|  | TGGTAATTAGTTTAAAAAAAATTAATGATTTCGACTCATTAGATTATGA---------------------- | 11.568 |
|  | ----------------------------------------------------TGTTCATAATTACCAACCA | 8.847 |
|  | TGGTAATTAGTTTAAAAAAAATTAATGATTTCGACTCATT------------------------------- | 8.775 |
|  | ----------------------------------------------------TGTTCATAATTACCAACC- | 6.577 |
|  | TGGTAATTAGTTTAAAAAAAATT------------------------------------------------ | 5.495 |
|  | TGGTAATTAGTTTAAAAAAAA-------------------------------------------------- | 4.703 |
| CH 1 | ------------------------------------------------------TGTTCATAATTACCAACCA | 15.707 |
|  | --------------------------------------------------------TTCATAATTACCAACCA | 4.495 |
|  | -------------------------------------------------------GTTCATAATTACCAACCA | 4.091 |
|  | ----------------------------------------------------------CATAATTACCAACCA | 3.889 |
|  | -----------------------------------------------------ATGTTCATAATTACCAACCA | 3.485 |
|  | --------------------------------------------------ATGATGTTCATAATTACCAACCA | 3.232 |
|  | ---------------------------------------------------TGATGTTCATAATTACCAACCA | 2.828 |
|  | ----------------------------------------------------GATGTTCATAATTACCAACCA | 2.424 |
|  | ---------------------------------------------------------TCATAATTACCAACCA | 2.323 |
|  | -----------------------------------------------ATGATGATGTTCATAATTACCAACCA | 1.818 |
| CH 2 | ------------------------------------------------------TGTTCATAATTACCAACCA | 3.596 |
|  | -----------------------------------------------------ATGTTCATAATTACCAACCA | 0.882 |
|  | -------------------------------------------------------GTTCATAATTACCAACCA | 0.858 |
|  | --------------------------------------------------------TTCATAATTACCAACCA | 0.766 |
|  | --------------------------------------------------ATGATGTTCATAATTACCAACCA | 0.696 |
|  | -----------------------------------------------ATGATGATGTTCATAATTACCAACCA | 0.650 |
|  | ---------------------------------------------------TGATGTTCATAATTACCAACCA | 0.603 |
|  | ----------------------------------------------------------CATAATTACCAACCA | 0.557 |
|  | ---------------------------------------------------------TCATAATTACCAACCA | 0.557 |
|  | ----------------------------------------------------GATGTTCATAATTACCAACCA | 0.534 |
| CH 3 | ------------------------------------------------------TGTTCATAATTACCAACCA | 42.145 |
|  | ------------------------------------------------------TGTTCATAATTACCAACC- | 13.686 |
|  | TGGTAATTAGTTTTAAAAAAAAATTAATGATTTCGACTCATTAGA---------------------------- | 8.006 |
|  | TGGTAATTAGTTTTAAAAAAAAATTAATGATTTCGACTCATTAGATTATGATGA------------------- | 6.647 |
|  | --------------------------------------------------------TTCATAATTACCAACCA | 5.680 |
|  | --------------------------------------------------ATGATGTTCATAATTACCAACCA | 4.804 |
|  | ---------------------------------------------------------TCATAATTACCAACCA | 4.683 |
|  | ----------------------------------------------------------CATAATTACCAACCA | 4.562 |
|  | -----------------------------------------------------ATGTTCATAATTACCAACCA | 4.290 |
|  | -------------------------------------------------------GTTCATAATTACCAACCA | 3.625 |
| HC 1 | ----------------------------------------------------TGTTCATAATTACCAACCA | 2.240 |
|  | ---------------------------------------------------ATGTTCATAATTACCAACCA | 2.005 |
|  | ------------------------------------------------ATGATGTTCATAATTACCAACCA | 1.875 |
|  | --------------------------------------------------------CATAATTACCAACCA | 1.615 |
|  | ------------------------------------------------------TTCATAATTACCAACCA | 1.432 |
|  | -------------------------------------------------------TCATAATTACCAACCA | 1.380 |
|  | -------------------------------------------------TGATGTTCATAATTACCAACCA | 1.198 |
|  | -----------------------------------------------------GTTCATAATTACCAACCA | 1.120 |
|  | ----------------------------TTTCGACTCATTAGATTATGATGATGTTCATAATTACCAACCA | 1.068 |
|  | --------------------------------------------TATGATGATGTTCATAATTACCAACCA | 0.938 |
| HC 2 | TGGTAATTAGTTTAAAAAAAATTAATGATTTCGACTCATTAGA---------------------------- | 12.635 |
|  | TGGTAATTAGTTTAAAAAAAATTAATGATTTCGACTCATTAGATTATGATGA------------------- | 8.498 |
|  | ----------------------------------------------------TGTTCATAATTACCAACCA | 7.315 |
|  | ----------TTTAAAAAAAATTAATGATTTCGACTCATTAGATTATGATGA------------------- | 3.350 |
|  | TGGTAATTAGTTTAAAAAAAATTAATGATTTCGACTCATTAGATT-------------------------- | 1.281 |
|  | ----------TTTAAAAAAAATTAATGATTTCGACTCATTAGATTATGATGATGTTCATAATTACCAACCA | 0.985 |
|  | TGGTAATTAGTTTAAAAAAAATTAATGATTTCGACTCATTAGATTA------------------------- | 0.714 |
|  | TGGTAATTAGTTTAAAAAAAATTAATGATTTCGACTCATTAGATTATGA---------------------- | 0.665 |
|  | TGGTAATTAGTTTAAAAAAAATTAATGATTTCGACTCATTAGAT--------------------------- | 0.640 |
|  | ------TTAGTTTAAAAAAAATTAATGATTTCGACTCATTAGATTATGATGA------------------- | 0.591 |
| HC 3 | ----------------------------------------------------TGTTCATAATTACCAACCA | 118.859 |
|  | TGGTAATTAGTTTAAAAAAAATTAATGATTTCGACTCATTAGATTATGATGA------------------- | 100.875 |
|  | ----------TTTAAAAAAAATTAATGATTTCGACTCATTAGATTATGATGA------------------- | 67.338 |
|  | TGGTAATTAGTTTAAAAAAAATTAATGATTTCGACTCATTAGA---------------------------- | 41.559 |
|  | ----------------------------------------------------TGTTCATAATTACCAACC- | 8.327 |
|  | -------------AAAAAAAATTAATGATTTCGACTCATTAGATTATGATGATGTTCATAATTACCAACCA | 6.654 |
|  | -------------------------------------------TTATGATGATGTTCATAATTACCAACCA | 6.502 |
|  | TGGTAATTAGTTTAAAAAAAATTAATGATTTCGACTCATTAGATT-------------------------- | 5.856 |
|  | ------TTAGTTTAAAAAAAATTAATGATTTCGACTCATTAGATTATGATGA------------------- | 5.741 |
|  | -----------------------------------------------------GTTCATAATTACCAACCA | 5.475 |
| HH 1 | --------------------------------------------------ATGATGTTCATAATTACCAACCA | 20.323 |
|  | ------------------------------------------------------TGTTCATAATTACCAACCA | 17.692 |
|  | -----------------------------------------------------ATGTTCATAATTACCAACCA | 17.047 |
|  | ----------------------------------------------------------CATAATTACCAACCA | 15.037 |
|  | ---------------------------------------------------TGATGTTCATAATTACCAACCA | 13.846 |
|  | --------------------------------------------------------TTCATAATTACCAACCA | 13.102 |
|  | -------------------------------------------------------GTTCATAATTACCAACCA | 12.903 |
|  | ---------------------------------------------------------TCATAATTACCAACCA | 12.581 |
|  | -----------------------------------------------ATGATGATGTTCATAATTACCAACCA | 11.042 |
|  | ----------------------------------------------------GATGTTCATAATTACCAACCA | 10.323 |
| HH 2 | ------------------------------------------------------TGTTCATAATTACCAACCA | 106.529 |
|  | TGGTAATTAGTTTTAAAAAAAAATTAATGATTTCGACTCATTAGATTATGATGA------------------- | 40.634 |
|  | TGGTAATTAGTTTTAAAAAAAAATTAATGATTTCGACTCATTAGA---------------------------- | 19.421 |
|  | ----------TTTTAAAAAAAAATTAATGATTTCGACTCATTAGATTATGATGA------------------- | 17.300 |
|  | --------------------------------------------------ATGATGTTCATAATTACCAACCA | 14.518 |
|  | -----------------------------------------------ATGATGATGTTCATAATTACCAACCA | 13.388 |
|  | -----------------------------------------------------ATGTTCATAATTACCAACCA | 13.278 |
|  | -------------------------------------------------------GTTCATAATTACCAACCA | 13.058 |
|  | ----------------------------------------------------------CATAATTACCAACCA | 12.562 |
|  | ---------------------------------------------------TGATGTTCATAATTACCAACCA | 11.983 |
| HH 3 | ------------------------------------------------------TGTTCATAATTACCAACCA | 20.739 |
|  | --------------------------------------------------ATGATGTTCATAATTACCAACCA | 11.756 |
|  | -----------------------------------------------ATGATGATGTTCATAATTACCAACCA | 10.813 |
|  | ---------------------------------------------------TGATGTTCATAATTACCAACCA | 8.743 |
|  | -----------------------------------------------------ATGTTCATAATTACCAACCA | 8.503 |
|  | -------------------------------------------------------GTTCATAATTACCAACCA | 8.096 |
|  | -------------------------------------------------GATGATGTTCATAATTACCAACCA | 7.431 |
|  | ----------------------------------------------------------CATAATTACCAACCA | 6.987 |
|  | ------------------------------------------------------TGTTCATAATTACCAACC- | 6.599 |
|  | ------------------------------------------------TGATGATGTTCATAATTACCAACCA | 6.451 |
| CF 1 | TGGTAATTAGTTTAAAAAAAAATTAATGATTTCGACTCATTAGATTATGATGA------------------- | 5.423 |
|  | -----------------------------------------------------TGTTCATAATTACCAACCA | 3.080 |
|  | ----------TTTAAAAAAAAATTAATGATTTCGACTCATTAGATTATGATGA------------------- | 2.820 |
|  | TGGTAATTAGTTTAAAAAAAAATTAATGATTTCGACTCATTAGA---------------------------- | 2.451 |
|  | ----------------------------------------------------ATGTTCATAATTACCAACCA | 2.299 |
|  | ---------------------------------------------------------CATAATTACCAACCA | 1.822 |
|  | ------------------------------------------------------GTTCATAATTACCAACCA | 1.779 |
|  | -------------------------------------------------------TTCATAATTACCAACCA | 1.692 |
|  | ----------------------------------------------ATGATGATGTTCATAATTACCAACCA | 1.649 |
|  | -------------------------------------------------ATGATGTTCATAATTACCAACCA | 1.497 |
| CF 2 | TGGTAATTAGTTTAAAAAAAAATTAATGATTTCGACTCATTAGATTATGATGA------------------- | 42.434 |
|  | -----------------------------------------------------TGTTCATAATTACCAACCA | 40.476 |
|  | TGGTAATTAGTTTAAAAAAAAATTAATGATTTCGACTCATTAGA---------------------------- | 25.608 |
|  | ----------TTTAAAAAAAAATTAATGATTTCGACTCATTAGATTATGATGA------------------- | 11.481 |
|  | -----------------------------------------------------TGTTCATAATTACCAACC- | 3.968 |
|  | TGGTAATTAGTTTAAAAAAAAATTAATGATTTCGACTCATTAGATT-------------------------- | 3.175 |
|  | TGGTAATTAGTTTAAAAAAAAATTAATGATTTCGACTCATTAGATTATGA---------------------- | 2.646 |
|  | ----------TTTAAAAAAAAATTAATGATTTCGACTCATTAGATTATGATGATGTTCATAATTACCAACCA | 2.063 |
|  | TGGTAATTAGTTTAAAAAAAAATTAATGATTTCGACTCATTAGAT--------------------------- | 1.905 |
|  | ------TTAGTTTAAAAAAAAATTAATGATTTCGACTCATTAGATTATGATGA------------------- | 1.799 |
| CF 3 | -----------------------------------------------------TGTTCATAATTACCAACCA | 1.933 |
|  | -------------------------------------------------ATGATGTTCATAATTACCAACCA | 1.681 |
|  | -------------------------------------------------------TTCATAATTACCAACCA | 1.345 |
|  | ----------------------------------------------------ATGTTCATAATTACCAACCA | 1.261 |
|  | ---------------------------------------------------------CATAATTACCAACCA | 1.092 |
|  | ------------------------------------------------GATGATGTTCATAATTACCAACCA | 0.966 |
|  | --------------------------------------------------------TCATAATTACCAACCA | 0.924 |
|  | ---------------------------------------------------GATGTTCATAATTACCAACCA | 0.924 |
|  | ------------------------------------------------------GTTCATAATTACCAACCA | 0.756 |
|  | --------------------------------------------------TGATGTTCATAATTACCAACCA | 0.756 |
| FF 1 | TGGTAATTAGTTTAAAAAAAAATTAATGATTTCGACTCATTAGATTATGATGA------------------- | 134.869 |
|  | TGGTAATTAGTTTAAAAAAAAATTAATGATTTCGACTCATTAGA---------------------------- | 53.670 |
|  | ----------TTTAAAAAAAAATTAATGATTTCGACTCATTAGATTATGATGA------------------- | 14.532 |
|  | TGGTAATTAGTTTAAAAAAAAATTAATGATTTCGACTCATTAGATT-------------------------- | 9.625 |
|  | TGGTAATTAGTTTAAAAAAAAATTAATGATTTCGACTCATTAGATTATGA---------------------- | 5.974 |
|  | TGGTAATTAGTTTAAAAAAAAATTAATGATTTCGACTCATTAGATTATGATGATG----------------- | 4.607 |
|  | ------TTAGTTTAAAAAAAAATTAATGATTTCGACTCATTAGATTATGATGA------------------- | 4.064 |
|  | -------------------------ATGATTTCGACTCATTAGATTATGATGA------------------- | 3.708 |
|  | TGGTAATTAGTTTAAAAAAAAATTAATGATTTCGACTCATT------------------------------- | 2.753 |
|  | -GGTAATTAGTTTAAAAAAAAATTAATGATTTCGACTCATTAGATTATGATGA------------------- | 2.734 |
| FF 2 | TGGTAATTAGTTTAAAAAAAAATTAATGATTTCGACTCATTAGATTATGATGA------------------- | 128.409 |
|  | TGGTAATTAGTTTAAAAAAAAATTAATGATTTCGACTCATTAGA---------------------------- | 42.149 |
|  | ----------TTTAAAAAAAAATTAATGATTTCGACTCATTAGATTATGATGA------------------- | 12.459 |
|  | -----------------------------------------------------TGTTCATAATTACCAACCA | 7.273 |
|  | TGGTAATTAGTTTAAAAAAAAATTAATGATTTCGACTCATTAGATT-------------------------- | 6.694 |
|  | TGGTAATTAGTTTAAAAAAAAATTAATGATTTCGACTCATTAGATTATGA---------------------- | 6.446 |
|  | TGGTAATTAGTTTAAAAAAAAATTAATGATTTCGACTCATTAGATTATGATGATG----------------- | 4.628 |
|  | ----------------------------------------------ATGATGATGTTCATAATTACCAACCA | 4.153 |
|  | ------TTAGTTTAAAAAAAAATTAATGATTTCGACTCATTAGATTATGATGA------------------- | 3.988 |
|  | -------------------------------------------------ATGATGTTCATAATTACCAACCA | 3.430 |
| FF 3 | TGGTAATTAGTTTAAAAAAAAATTAATGATTTCGACTCATTAGATTATGATGA------------------- | 67.222 |
|  | TGGTAATTAGTTTAAAAAAAAATTAATGATTTCGACTCATTAGA---------------------------- | 22.488 |
|  | ----------TTTAAAAAAAAATTAATGATTTCGACTCATTAGATTATGATGA------------------- | 11.981 |
|  | TGGTAATTAGTTTAAAAAAAAATTAATGATTTCGACTCATTAGATTATGA---------------------- | 4.082 |
|  | TGGTAATTAGTTTAAAAAAAAATTAATGATTTCGACTCATTAGATT-------------------------- | 4.034 |
|  | TGGTAATTAGTTTAAAAAAAAATTAATGATTTCGACTCATTAGATTATGATGATG----------------- | 3.237 |
|  | ------TTAGTTTAAAAAAAAATTAATGATTTCGACTCATTAGATTATGATGA------------------- | 2.585 |
|  | -----------------------------------------------------TGTTCATAATTACCAACCA | 2.246 |
|  | TGGTAATTAGTTTAAAAAAAAATTAATGATTTCGACTCATTAGATTA------------------------- | 2.246 |
|  | TGGTAATTAGTTTAAAAAAAAATTAATGATTTCGACTCATTAGATTATGATGAT------------------ | 2.150 |
