# Supplementary material for: Mitochondrial tRNA-Derived Fragments as Candidate Metastasis-Modifying RNA
Source: Cancer Res Commun. 2026 Aug 28;6(8):2017–25. doi: 10.1158/2767-9764.CRC-26-0360 (PMC13522972; doi:10.1158/2767-9764.CRC-26-0360)
Supplement: Supplementary file 4 [file crc-26-0360_supplementary_table_2_suppst2.docx]

**Supplementary Table 2: AlphaFold3 predictive modeling of D-T Loop interactions of mt-TR.** Predictive bonding and distances between the center of nucleotides involved in interactions between the D and T loop. Notes include specific atoms of each nucleotide involved in hydrogen bonding. Notably, predicted interactions vary across the different mtDNA backgrounds due to nucleotide polymorphisms.

|  | **Nucleotide & Position** | **Distance (Å)** | **Notes** |
| --- | --- | --- | --- |
| **CC & HC** | A15-U55 | 11.46 | No bonding |
|  | A16-G54 | 8.18 | Two hydrogen bonds (A16 N3 – G54 ‘O2; A16 N3 – G54 O3’) |
|  | A16-U55 | 7.83 | No bonding |
|  | A17-U53 | 11.11 | No bonding |
|  | A17-G54 | 10.93 | No bonding |
|  | U44-G54 | 6.6 | Hydrogen bond (U44 O4 - G54 ‘O2) |
|  | U44-U55 | 11.76 | No bonding |
| **HH & CH** | A15-U57 | 6.97 | Pi interaction |
|  | A16-G56 | 12.36 | No bonding |
|  | A16-U57 | 5.93 | No bonding |
|  | A17-U55 | 12.96 | No bonding |
|  | A17-G56 | 12.22 | No bonding |
|  | U46-G56 | 14.21 | No bonding |
|  | U46-U57 | 10.38 | Hydrogen bond (U46 N3 - U57 O4) |
| **FF & CF** | A15-U56 | 7.47 | Hydrogen bond (A15 O2' - U56 O2) |
|  | A16-G55 | 11.26 | No bonding |
|  | A16-U56 | 7.42 | Hydrogen bond (A16 N7 - U56 O2’) |
|  | A17-U54 | 6.87 | Pi interaction and Hydrogen bond (A17 O2’ – U54 O2’) |
|  | A17-G55 | 6.93 | Pi interaction (opposite to U54 Pi interaction) |
|  | U45-G55 | 12.73 | No bonding |
|  | U45-U56 | 8.56 | No bonding |
